# River damming enhances ecological functional stability of planktonic microorganisms

**DOI:** 10.3389/fmicb.2022.1049120

**Published:** 2022-11-30

**Authors:** Wanzhu Li, Baoli Wang, Na Liu, Meiling Yang, Cong-Qiang Liu, Sheng Xu

**Affiliations:** ^1^Institute of Surface-Earth System Science, School of Earth System Science, Tianjin University, Tianjin, China; ^2^Tianjin Bohai Rim Coastal Earth Critical Zone National Observation and Research Station, Tianjin, China

**Keywords:** river damming, planktonic microorganism, microbial taxonomy, functional gene, ecological functional stability

## Abstract

Planktonic microorganisms play an important role in maintaining the ecological functions in aquatic ecosystems, but how their structure and function interrelate and respond to environmental changes is still not very clear. Damming interrupts the river continuum and alters river nutrient biogeochemical cycling and biological succession. Considering that river damming decreases the irregular hydrological fluctuation, we hypothesized that it can enhance the ecological functional stability (EFS) of planktonic microorganisms. Therefore, the community composition of planktonic bacteria and archaea, functional genes related to carbon, nitrogen, sulfur, and phosphorus cycling, and relevant environmental factors of four cascade reservoirs in the Pearl River, Southern China, were investigated to understand the impact of damming on microbial community structure and function and verify the above hypothesis. Here, the ratio of function to taxa (F:T) based on Euclidean distance matrix analysis was first proposed to characterize the microbial EFS; the smaller the ratio, the more stable the ecological functions. The results showed that the reservoirs created by river damming had seasonal thermal and chemical stratifications with an increasing hydraulic retention time, which significantly changed the microbial structure and function. The river microbial F:T was significantly higher than that of the reservoirs, indicating that river damming enhances the EFS of the planktonic microorganisms. Structural equation modeling demonstrated that water temperature was an important factor influencing the relationship between the microbial structure and function and thus affected their EFS. In addition, reservoir hydraulic load was found a main factor regulating the seasonal difference in microbial EFS among the reservoirs. This study will help to deepen the understanding of the relationship between microbial structure and function and provide a theoretical basis of assessing the ecological function change after the construction of river damming.

## Introduction

Microorganisms play a key role in driving elemental biogeochemical cycles. In the long-term evolution, *via* lateral gene transfer, a set of core genes remain relatively stable among different microbial taxa to code the major redox reactions related to the biogeochemical cycles of nutrients (Falkowski et al., 2008). Meanwhile, microorganisms have evolved enormous species diversity as well. Therefore, the relationship between structure and function of microbial community and relevant mechanisms maintaining ecosystem stability have been hot issues in ecology (Sutherland et al., 2013). Nowadays microbial community assembly is well known to be affected not only by environmental filtering and biotic interaction (that are deterministic processed based on niche theory), but also by some random events, including drift, extinction, and mutation (that are stochastic processes based on neutral theory; Hubbell, 2006; Stegen et al., 2012; Zhou et al., 2014). Microbial functional traits are measurable characteristics that impact their fitness and performance and can be recognized across the biological hierarchy of gene, organism, guild, and community (Yang, 2021). The traits are widely used to understand the ecological functions (e.g., resource acquisition, growth, reproduction and survival) of microorganisms (Litchman and Klausmeier, 2008; Martini et al., 2021). Compared with microbial community assembly, the functional traits show more strongly relevant to deterministic processes. For example, in the *Tara* Ocean, environmental conditions can predict functional traits of bacterial and archaeal communities very well, but weakly predict their taxonomic composition (Longhi and Beisner, 2010; Louca et al., 2016). As such, environmental conditions can affect ecosystem functioning by influencing either microbial taxonomic composition or microbial functional traits (Allison and Martiny, 2008; Abonyi et al., 2018; Wagg et al., 2019; Hong et al., 2022). Therefore, it is one-sided to predict ecosystem functioning by just considering the response of microbial taxonomic composition (or functional traits) to environmental changes, and only jointly investigating the structure and function can systematically understand ecosystem functional redundancy and/or resilience to perturbations (Martini et al., 2021).

Since the Industrial Revolution, anthropogenic activities, as a new geological force, have become an important power shaping the earth’s environment and surface evolution (Lewis and Maslin, 2015). Nowadays damming is the most significant anthropogenic disturbance to rivers, and approximately 70% of the world’s rivers have been dammed (Grill et al., 2019), which has greatly changed the ecological environment of rivers. Dams interrupt the river continuum, increase the retention time of water and nutrients, and alter nutrient cycling and biological succession (Wang B. et al., 2018; Wu et al., 2019; Maavara et al., 2020; Xiao et al., 2021). Reservoirs created by damming are influenced by anthropogenic regulation with their own specific ecological characteristics, such as seasonal thermal, chemical, and biological stratifications, and damming effects become a hot ecological issue (Maavara et al., 2020; Wang B. et al., 2022). As for the aspect of river planktonic microorganisms, many studies focused on the microbial biogeographic distribution pattern and its controlling mechanisms, rather than microbial functional traits (Wang X. et al., 2018; Nyirabuhoro et al., 2020; Yang et al., 2020; Lu et al., 2022). As such, it is unclear for the relationship between structure and function of microbial community in dammed rivers nowadays. Considering that dam reservoirs can regulate the disordered hydrological rhythms of rivers, we hypothesized that river damming can enhance the ecological functional stability (EFS) of planktonic microorganisms.

Therefore, the community composition of planktonic bacteria and archaea, functional genes related to carbon (C), nitrogen (N), sulfur (S), and phosphorus (P) cycling, and relevant environmental factors of four cascade reservoirs with different hydrological conditions and their inflowing and released waters in the Pearl River, Southern China, were investigated to verify the above hypothesis. The main aims of this study are to identify the environmental factors affecting the microbial community structure and function in dammed rivers and to understand the impact of damming on the planktonic microbial EFS. This study will help to deepen the understanding of the relationship between microbial community structure and function and provide a theoretical basis on assessing the river ecological function change after the construction of dam reservoirs.

### A proposed conceptual framework

**Figure 1:**
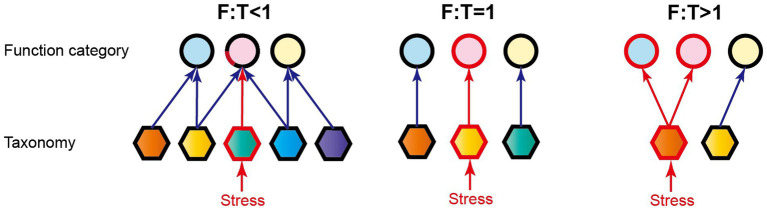
Three scenarios of the relationship between microbial structure and function. Circles and hexagons represent the function categories and taxonomies, respectively. The arrows between the above two indicate the ecological function performed by microorganisms. Difference color fillings indicate different types of function categories or taxonomies. F:T, the ratio of functional pathway to microbial taxa-based Euclidean distance. The red lines and frames indicate the influenced portions by environmental stresses, and thus F:T < 1 means strong ecological functional stability (the other details refer to the text).

Functional genes are an important parameter characterizing microbial functional traits at a molecular level, and different microbial species can have genes with the same or similar functions. For example, both ammonia-oxidizing archaea (e.g., Thaumarchaeota and Crenarchaeota) and bacteria (e.g., Proteobacteria and Bacteroidetes) contain *amoA* gene that is involved in N-cycling (Yakimov et al., 2011; Auguet et al., 2012). These ecological incoherencies lead to functional similarity among taxonomically distinct microorganisms and beget functional redundancy to buffer ecosystem functioning against species loss, finally maintaining the stability of ecosystems (Allison and Martiny, 2008; Louca et al., 2018; Yang, 2021). Based on the theory of functional redundancy, we proposed a dimensionless ratio of function (F) to taxa (T) based on Euclidean distance matrix analysis (i.e., F:T) for quantifying microbial EFS in reservoir ecosystems. The F:T ratio represents the degree of relative change for microbial function in comparison with structure, *via* comparing Euclidean distance of function (i.e., functional composition) with that of microbial taxa (i.e., composition of archaea and/or bacteria; Figure 1). Obviously, the synchronous variations in function and community structure mean F:T = 1. When F:T is less than 1, the larger variation in microbial taxa is compared with the smaller variation in function, indicating that multiple taxa govern the same or similar ecological functions. These replaceable microorganisms can take turns to perform the ecological functions against the disturbance of species loss. The disturbance under this situation will have slight influence on ecological functions, and ecosystems are thus ensured strong stability (Louca et al., 2018; Biggs et al., 2020). The smaller the ratio, the more stable the ecological functions. When F:T is more than 1, the smaller variation in microbial taxa in comparison with the larger variation in function suggests that species loss could strongly influence multiple related ecological functions, and the stability of ecosystems is relatively low (Figure 1).

## Materials and methods

### Study area and sampling

**Figure 2:**
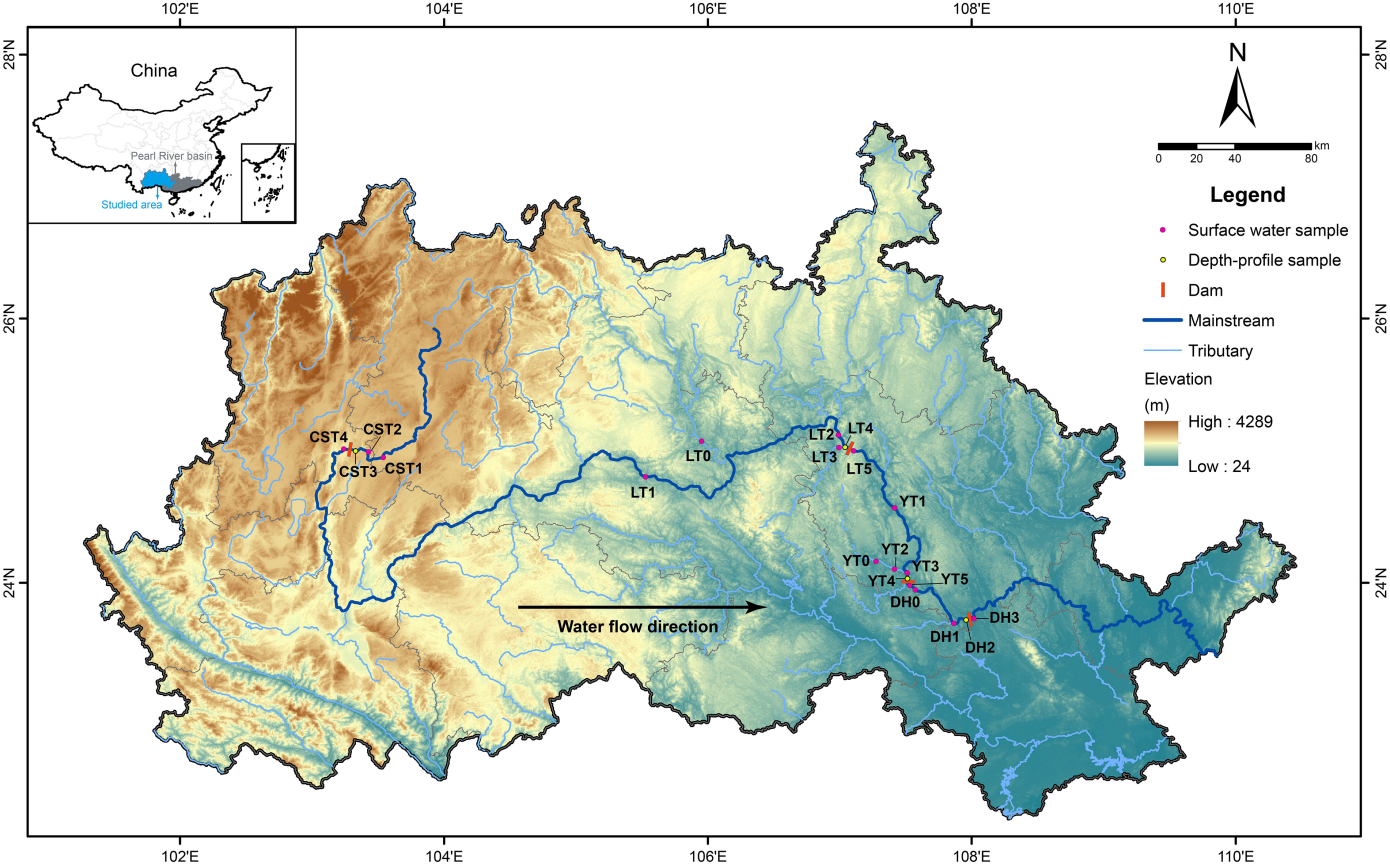
Sampling sites and locations in the Pearl River basin, Southern China. The full names of reservoirs refer to the text.

The Pearl River in southern China has a subtropical monsoon climate, with average annual temperature and precipitation of 14–22°C and 1,200–2,200 mm, respectively. The inflowing, reservoir, and released waters were sampled in August and December 2019 for four hydropower reservoirs (Figure 2), of which the Chaishitan reservoir (CST) is the leading reservoir in the upper reaches of the Pearl River, and the Longtan reservoir (LT), Yantan reservoir (YT), and Dahua reservoir (DH) are located successively on the river. Water samples were taken at different depths: CST at the depth of 0, 5, 10, 15, 30, and 55 m, LT at the depth of 0, 5, 10, 15, 30, 60, 80, and 140 m, YT at the depth of 0, 5, 10, 15, 30, and 50 m, and DH at the depth of 0, 5, 10, 15, and 25 m, respectively. Other water samples were obtained from the surface waters. The detailed information of sampling sites was listed in Supplementary Table 1. Water temperature (WT), pH, and dissolved oxygen concentration (DO) were determined *in situ* by an automated multiparameter profiler (YSI, EXO1, United States) with precorrection. Photosynthetic efficiency (i.e., effective quantum yield, yield) and chlorophyll a concentration (Chl *a*) were measured by a phytoplankton analyzer (Phyto-PAM, Walz, Germany). Water samples were filtered through 0.45 μm Millipore cellulose acetate membranes, and the filtered waters were collected in 60 ml plastic bottles and stored at 4°C for measuring nutrient concentrations. The samples of 16S rRNA and functional genes were collected using 0.22 μm Millipore cellulose acetate membranes and stored at-20°C for the extraction of DNA; the filtered waters were collected in 15 ml centrifuge tubes and stored at 4°C for measuring cation and anion concentrations and dissolved strontium (Sr) concentration. All samples were treated within 12 h after sampling.

### Measurement of physical and chemical parameters

Cation and anion concentrations (e.g., Na^+^, K^+^, Mg^2+^, Ca^2+^, Cl^−^, SO_4_^2−^, and NO_3_^−^) were determined by ion chromatography (ICS-5000, Thermo Fisher, United States). The Sr concentration was determined by inductively coupled plasma mass spectrometer (ICP-MS, Agilent, United States) with 0.01 mg L^−1^ detection limit. Nutrient concentrations, including nitrate nitrogen (NO_3_-N), nitrite nitrogen (NO_2_-N), ammonia nitrogen (NH_4_-N), phosphate phosphorus (PO_4_-P), and dissolved silicon (DSi), were measured by Skalar (SAN++, the Netherlands) with detection limits of 0.01 mg L^−1^, 0.005 mg L^−1^, 0.02 mg L^−1^, 0.02 mg L^−1^, and 0.02 mg L^−1^, respectively. The concentration of dissolved inorganic nitrogen (DIN) was the sum of NO_3_-N, NO_2_-N, and NH_4_-N concentrations. The concentration of dissolved inorganic phosphorus (DIP) was referred to PO_4_-P concentration. Total alkalinity (ALK) was determined by titration with HCl. The dissociation constants for carbonic acid were calculated by the temperature and corrected by ionic strength (Stumm and Morgan, 1983; Maberly, 1996). The concentrations of CO_2_, HCO_3_^−^, and CO32-were calculated using ALK, pH, and corrected dissociation constants. The concentration of dissolved inorganic carbon (DIC) is the sum of the above three ionic concentrations.

### Gene sequencing and qPCR for specific genes

The total DNA of sample was extracted by E.Z.N.ATM Water DNA Kit (OMEGA, United States) according to manufacturer’s protocols. The concentration and purity of DNA were determined by Multiskan GO (Thermo Fisher, United States). The details for DNA extraction are referred to Wang et al. (2021). The V4 region of 16S rRNA sequencing for archaea and bacteria was performed on Illumina NovaSeq 6,000 platform at Meige Technology Co., Ltd., Guangzhou, China. The 515F (GTGCCAGCMGCCGCGGTAA) and 806R (GGACTACHVGGGTWTCTAAT) were used as primers for bacteria. The Arch340F (CCCTAYGGGGYGCASCAG), Arch1000F (GGCCATGCACYWCYTCTC), Uni519F (CAGYMGCCRCGGKAAHACC), and Arch806R (GGACTACNSGGGTMTCTAAT) were used as primers for archaea. Raw fastq data were quality-filtered using Trimomatic (Bolger et al., 2014) to remove contaminating adaptors and short length reads (<200 bp). The overlapping paired-end reads with sequence mismatching < 5 bp and alignment similarity > 90% were merged using FLASH (Magoč and Salzberg, 2011). Operational taxonomic units (OTUs) were clustered in UPARSE software at 97% consistency level (Edgar, 2013). Singleton and doubleton OTUs representing sequencing errors were removed, and the representative OTUs with the highest occurrence frequency were assigned using Silva, RDP, and Greengenes (Lan et al., 2012). To equalize sequencing depth, each sample was rarefied to the minimum sequencing depth, and sequence normalization was performed using MOTHUR v.1.33.3 (Schloss et al., 2009). The raw data of 16S rRNA sequencing was deposited in NCBI SRA database with the accession numbers of PRJNA874581, PRJNA874587, PRJNA874586, and PRJNA874582.

The extracted particulate DNA was subjected to real-time quantitative polymerase chain reaction (qPCR) to quantify the functional gene abundance related to C-, N-, P-, and S-cycling. The qPCR for specific genes was conducted by a commercial service at Meige Technology Co., Ltd., Guangzhou, China. The ranges of qPCR efficiency were 95%–110%.

### Statistical analysis

Plotting was conducted by Origin 2021. The statistical analyses were performed by R software [version 4.1.2, (R Core Team, 2021)]. Pearson’s correlation analyses were conducted using “corrplot” libraries. Shannon-wiener index was conducted with the “vegan” libraries. Spearman’s correlation analyses were performed using “corrplot” libraries. Mantel test was used for correlational analysis according to the *r* and significance level *value of p* of the two matrices. Structural equation modeling was conducted using AMOS software (SPSS). Co-occurrence networks were constructed using the “WGCNA” libraries, and were visualized with “Gephi 0.9.6” software. The pairwise Pearson’s correlations between parameters were calculated, with a correlation coefficient > 0.6 and a *p*-value <0.05 (Benjamini and Hochberg adjusted) being considered as a valid relationship to further calculate topological features of a network. Euclidean distance was performed using “vegan” libraries. Based on the relative abundance of functional pathways and dominant phyla of bacteria and archaea, Euclidean distance matrix of all samples were calculated, and then the averages of Euclidean distances of one sample in relation to other samples in microbial taxa (i.e., bacteria and archaea taxa, T_mic_), functional pathway (i.e., F), bacteria taxa (T_bac_), and archaea taxa (T_arc_) were obtained. The solar radiation data of sampling site was obtained by the agrometeorological data.^1^ The *t*-tests were used to determine the significant differences within the 95% confidence interval between different groups and conducted by IBM SPSS Statistics 23.

## Results

### Physical and chemical parameters

The average WT, pH, DO, and Chl *a* were 22°C, 7.8, 6.9 mg L^−1^, and 7.4 μg L^−1^, respectively. Phytoplankton yield ranged from 0.15 to 0.46, with an average of 0.32. The upstream reservoir (i.e., CST) showed the lowest average WT but the highest average Chl *a* (Table 1). There were obvious differences in WT among the reservoirs but insignificant difference between the inflowing and reservoir waters. However, the DO of inflowing waters was obviously higher than that of reservoir waters (Table 1). Stratifications of WT, pH, DO, and Chl *a* occurred in CST and LT with long hydraulic retention time in August, but not in YT and DH (Supplementary Figure 1 and Supplementary Table 2). The CST showed higher DIC, DIN, and DIP concentration but lower DSi concentration than other reservoirs. Overall, the inflowing waters exhibited similar nutrient concentrations to the reservoir waters. The molar ratio of dissolved Ca to Sr concentrations (Ca/Sr) can indicate the geological source of solutes in rivers (Jiang and Ji, 2011). The average Ca/Sr was 719.1, indicating that the solutes in the river were mainly controlled by carbonate weathering. The Ca/Sr of CST was slightly higher than that of other reservoirs, suggesting that the contribution of silicate weathering in the upstream was greater than that in the downstream (Qiu et al., 2022).

**Table 1:** The averages and ranges of physical and chemical factors of the studied reservoirs in the Pearl River.

Factor		CST	LT	YT	DH	Inflowing water	Reservoir
WT (°C)	Min-Max	13.3–28.0	16.7–29.5	20.5–29.4	20.4–24.6	28.1–15.4	13.3–29.5
	Aver (SD)	19.4 (4.7)	22.5 (3.9)	23.0 (2.5)	22.4 (2.1)	22.2 (3.6)	22.0 (3.7)
pH	Min-Max	7.1–9.0	7.2–8.8	7.4–8.5	7.8–7.9	7.7–8.2	7.1–9.0
	Aver (SD)	7.6 (0.6)	7.9 (0.4)	7.8 (0.3)	7.8 (0.0)	8.0 (0.2)	7.8 (0.4)
DO (mg L^−1^)	Min-Max	0.2–15.5	0.9–11.2	6.6–10.9	6.6–7.9	6.9–18.3	0.2–15.5
	Aver (SD)	4.5 (4.7)	6.2 (2.8)	7.6 (1.1)	7.7 (0.3)	8.8 (3.2)	6.6 (2.8)
Chl *a* (μg L^−1^)	Min-Max	2.9–72.0	2.3–17.4	2.3–13.3	2.1–3.0	2.5–22.4	2.1–72.0
	Aver (SD)	24.6 (27.5)	7.2 (5.4)	4.4 (2.8)	2.5 (0.3)	5.9 (5.5)	8.6 (14.5)
DIC (mmol L^−1^)	Min-Max	2.0–4.7	1.9–3.3	2.2–3.3	2.9–3.0	2.6–3.6	1.9–4.7
	Aver (SD)	3.2 (0.8)	2.8 (0.4)	3.0 (0.2)	2.9 (0.1)	3.0 (0.3)	3.0 (0.5)
DIN (μmol L^−1^)	Min-Max	55.2–517.8	98.9–166.8	56.2–218.2	123.7–161.0	63.8–435.2	55.2–517.8
	Aver (SD)	365.0 (146.0)	133.1 (16.6)	143.2 (31.3)	144.2 (14.8)	204.8 (109.6)	187.3 (115.5)
DIP (μmol L^−1^)	Min-Max	0.3–4.2	0.3–1.4	0.3–1.0	0.3–1.4	0.3–1.7	0.3–4.2
	Aver (SD)	1.9 (1.5)	0.8 (0.5)	0.6 (0.3)	0.6 (0.3)	0.8 (0.5)	1.0 (0.9)
DSi (μmol L^−1^)	Min-Max	44.7–252.9	97.1–292.1	92.9–282.4	93.7–262.3	30.2–337.3	44.7–292.1
	Aver (SD)	124.0 (76.7)	174.0 (79.1)	175.8 (85.1)	177.6 (87.2)	171.7 (100.5)	164.5 (82.6)
SO_4_^2−^ (μmol L^−1^)	Min-Max	10.8–670.8	7.3–366.6	31.4–375.8	288.9–338.2	65.1–740.8	8.8–805.0
	Aver (SD)	514.0 (176.2)	288.3 (113.9)	275.8 (112.0)	313.5 (23.8)	404.4 (174.3)	405.7 (179.0)
Ca/Sr	Min-Max	1041.3–1724.1	276.6–549.2	398.4–904.1	476.0–569.2	305.7–2491.4	276.6–1724.1
	Aver (SD)	1460.5 (222.0)	434.8 (82.9)	526.1 (110.6)	510.4 (29.5)	866.7 (670.0)	696.5 (425.4)

WT, water temperature; DO, dissolved oxygen concentration; Chl *a*, chlorophyll a concentration; DIC, dissolved inorganic carbon; DIN, dissolved inorganic nitrogen; DIP, dissolved inorganic phosphorus; DSi, dissolved silicon; Ca/Sr, the molar ratio of dissolved Ca to Sr concentrations. The full names of the reservoirs refer to the text.

### Microbial community structure and function

**Figure 3:**
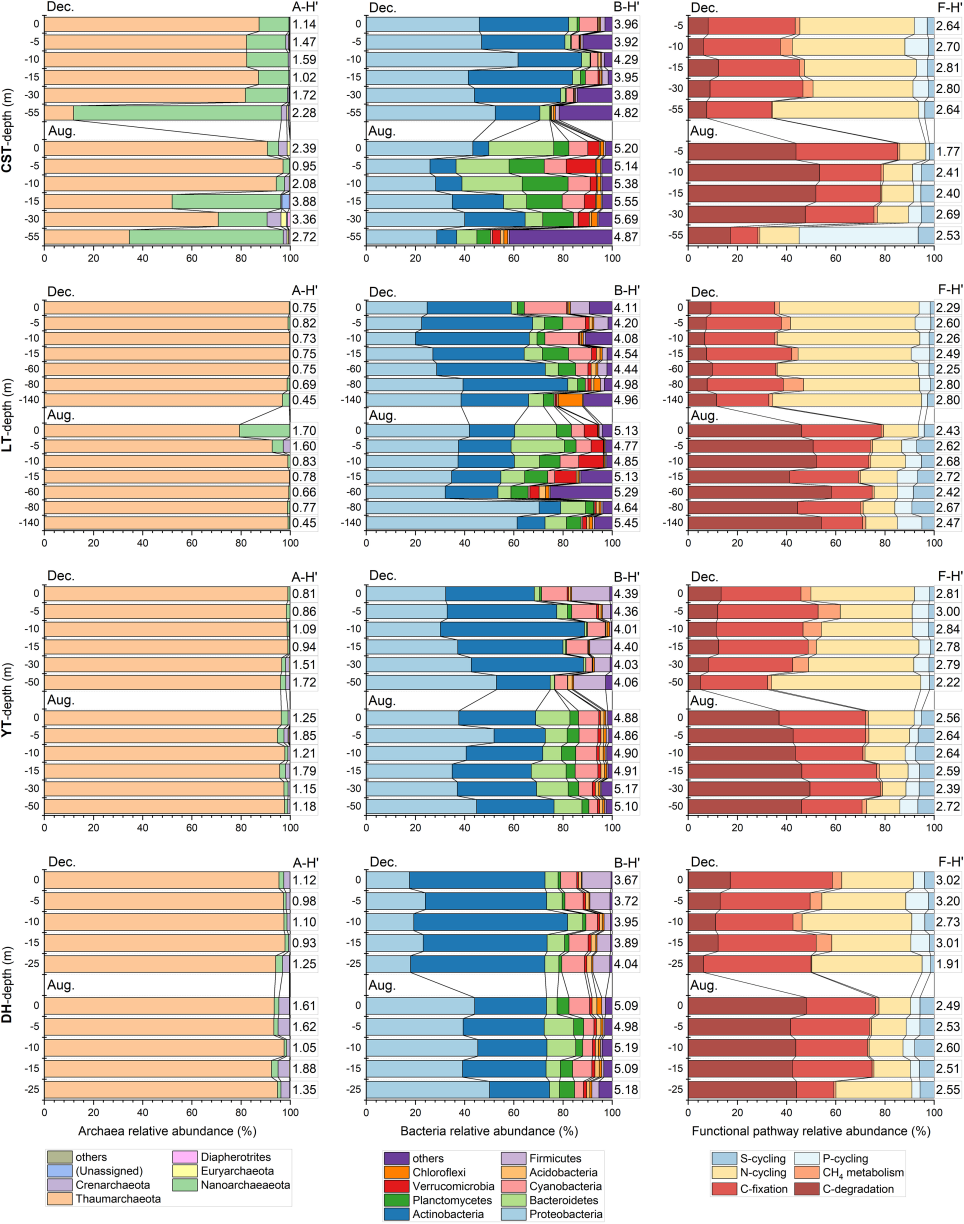
The planktonic archaea and bacteria community composition and relative abundance of functional pathway in August and December in the water profiles of the studied reservoirs. A-H′, planktonic archaea community diversity; B-H′, planktonic bacteria community diversity; F-H′: functional diversity. Taxonomic groups with a relative abundance less than 1% were integrated as “others.” The full names of the reservoirs refer to the text.

The maximum number of planktonic archaea OTUs yielded by high-throughput sequencing was 76,545. All archaea OTUs were clustered into 9 phyla. The top two archaea phyla were Thaumarcheaota and Nanoarchaeaeota. Thaumarcheaota was the absolute dominant phylum in the reservoirs except CST. The first two dominant phyla changed greatly in the water profiles of CST and LT but varied small in the water profiles of YT and DH. The planktonic archaeal diversity index (i.e., Shannon-Wiener index, A-H′) ranged from 0.45 to 4.47, with an average of 1.43 (Figure 3). The A-H′ of inflowing waters (1.98 ± 1.22) was higher than that of reservoir waters (1.32 ± 0.66). The maximum number of planktonic bacteria OTUs yielded by high-throughput sequencing was 661,239. All bacteria OTUs were clustered into 57 phyla. The top four bacteria phyla were Proteobacteria, Actinobacteria, Bacteroidetes, and Planctomycetes. The bacterial community structure was different in time and space. For example, the bacterial community composition in the inflowing waters of CST was obviously different from that in the reservoir (Supplementary Figure 2). In the reservoir profiles, the first four dominant phyla had great difference. In addition, the bacteria phyla with relative abundance less than 10% had great changes in the water profiles of CST and LT but had not in YT and DH. The planktonic bacterial diversity index (i.e., Shannon-Wiener index, B-H′) ranged from 3.36 to 5.69, with an average of 4.60 (Figure 3). The B-H′ of inflowing waters (4.71 ± 0.75) was slightly higher than that of reservoir waters (4.59 ± 0.54).

**Figure 4:**
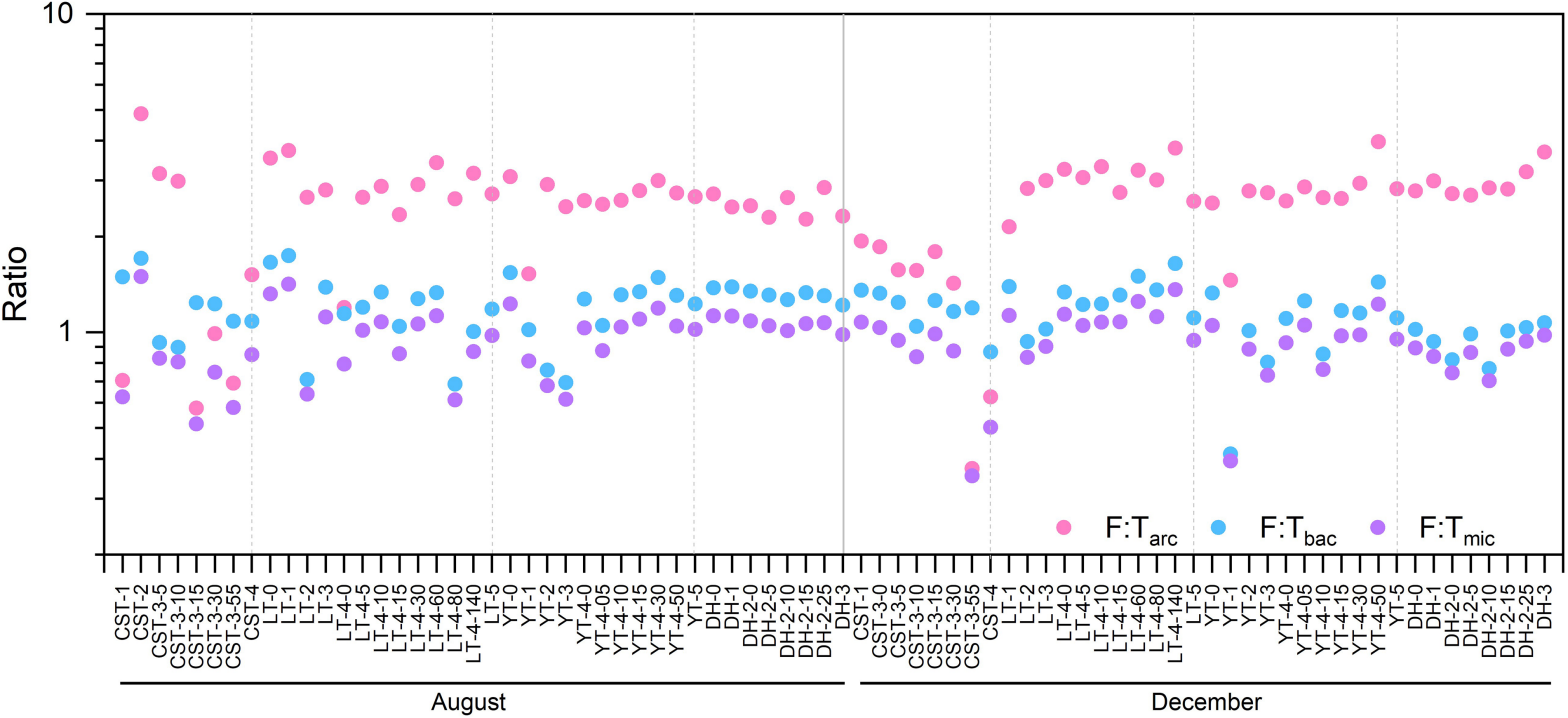
The spatio-temporal distribution of the ratios in the studied reservoirs. The full names of the reservoirs refer to the text. F:T_arc_, the ratio of functional pathway to archaea taxa-based Euclidean distance; F:T_bac_, the ratio of functional pathway to bacteria taxa-based Euclidean distance; F:T_mic_, the ratio of functional pathway to microbial taxa-based Euclidean distance.

**Figure 5:**
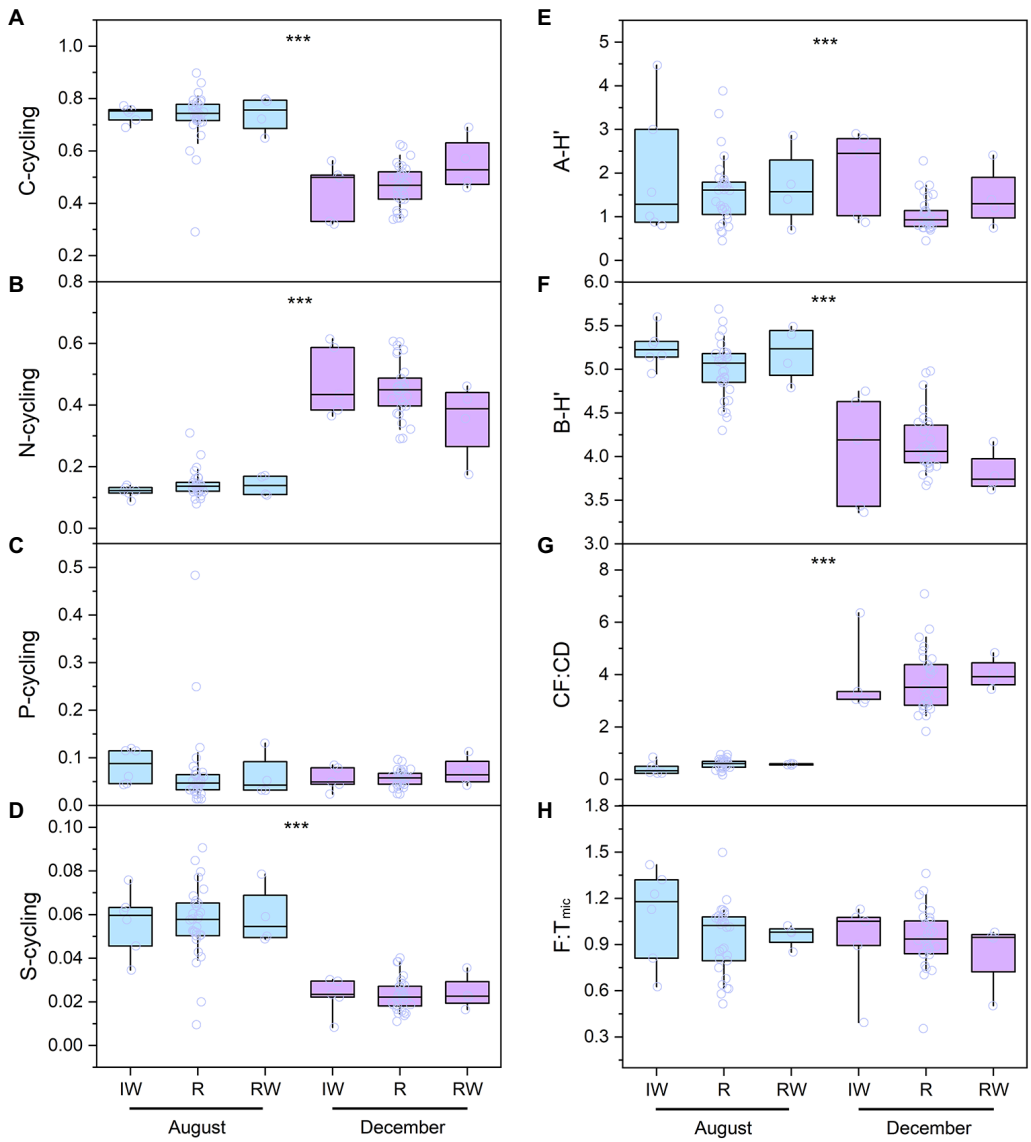
The spatio-temporal distribution of the microbial taxa and function in the studied reservoirs **(A-H)**. The boxes and whiskers indicate the 25th and 75th and 10th and 90th percentiles, respectively. The central lines indicate the medians. Blue and purple boxes represent sampling in August and December, respectively. The full names of the reservoirs refer to the text. IW, inflowing waters; R, reservoir; RW, released waters; CF:CD, the ratio of carbon fixation to carbon degradation; A-H′, planktonic archaea community diversity; B-H′, planktonic bacteria community diversity; F:T_mic_, the ratio of functional pathway to microbial taxa-based Euclidean distance. ^***^indicate the significant difference at the levels of 0.001 conducted by *t*-test.

In this study, C-cycling included three functional pathways [i.e., C-fixation (CF), C-degradation (CD), and CH_4_ metabolism] and had 36 functional genes, with an average of 3.5 × 10^5^ copies L^−1^. N-cycling included 22 functional genes, with an average of 2.4 × 10^5^ copies L^−1^. P-cycling included 8 functional genes, with an average of 2.1 × 10^5^ copies L^−1^. S-cycling included 5 functional genes, with an average of 1.9 × 10^5^ copies L^−1^. Functional diversity (i.e., Shannon Wiener index, F-H′) ranged from 1.77 to 3.20, with an average of 2.60 (Figure 3). The F-H′ of inflowing waters (2.55 ± 0.25) was similar to that of reservoir waters (2.57 ± 0.31). The F:T_arc_ ratios were higher than the ratios of F:T_bac_ and F:T_mic_ in the reservoirs except CST in time and space (Figure 4). The C-, N-and S-cycling pathways and microbial diversity had significant seasonal differences (*p* < 0.001, *t*-test, Figures 5A–F). The CF:CD ratio was higher in December than in August (Figure 5G). In general, the F:T_mic_ ratios in inflowing waters (1.01 ± 0.3) were slightly higher than that in reservoir waters (0.94 ± 0.21; Figure 5H). However, in the LT with long hydraulic retention time, the F:T_mic_ ratio of inflowing waters was significantly higher than that of reservoir waters (*p* = 0.01, *t*-test).

### Interaction among the environmental factors, community, and functional pathway

**Figure 6:**
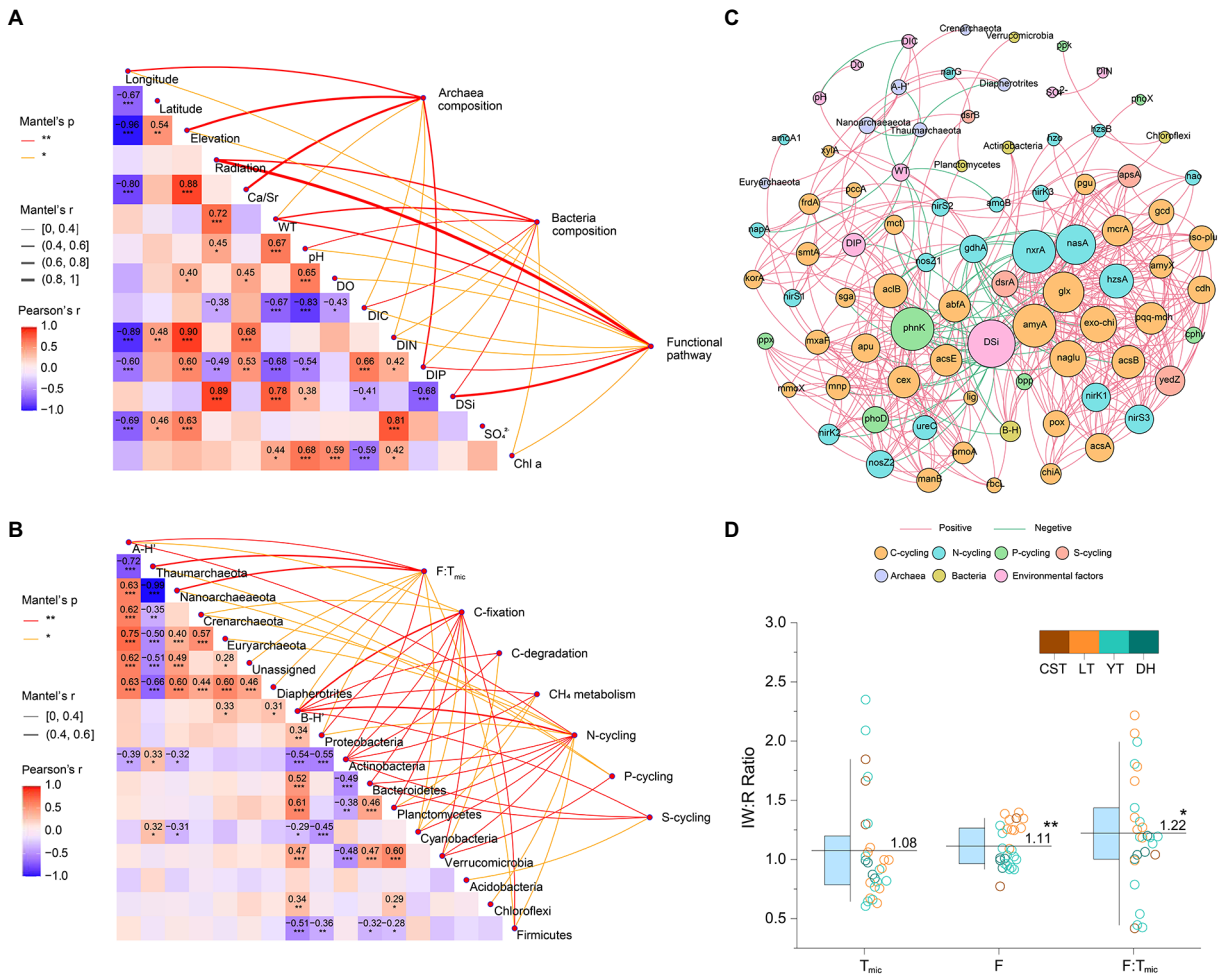
**(A)** Correlations among environmental factors, community composition of planktonic bacteria and archaea and functional pathway for surface water samples (*n* = 36). Community composition and functional pathway are related to each environmental factor by performing a Mantel test. **(B)** Correlations between community composition of planktonic bacteria and archaea and functional pathway (*n* = 78). Pairwise comparisons within community composition are displayed with a color gradient to denote Pearson’s correlation coefficients. Functional pathways are related to each community parameter by performing a Mantel test. Asterisks (^***^, ^**^, and ^*^) indicate the significance levels (0.001, 0.01 and 0.05, respectively). A-H’, planktonic archaea community diversity; B-H’, planktonic bacteria community diversity. **(C)** Co-occurrence networks of the planktonic bacteria and archaea community and functional genes. The size of each node is proportional to the number of connections. **(D)** The parameter ratio of inflowing to reservoir waters. IW, inflowing waters; R, reservoir; F, functional pathway based-Euclidean distance; T_mic_, microbial taxa based-Euclidean distance. The different colors indicate different reservoirs. The boxes and whiskers indicate the 25th and 75th and 10th and 90th percentiles, respectively. The central lines indicate the averages. ^**^ and ^*^ were significant difference at the levels of 0.01 and 0.05, respectively.

**Figure 7:**
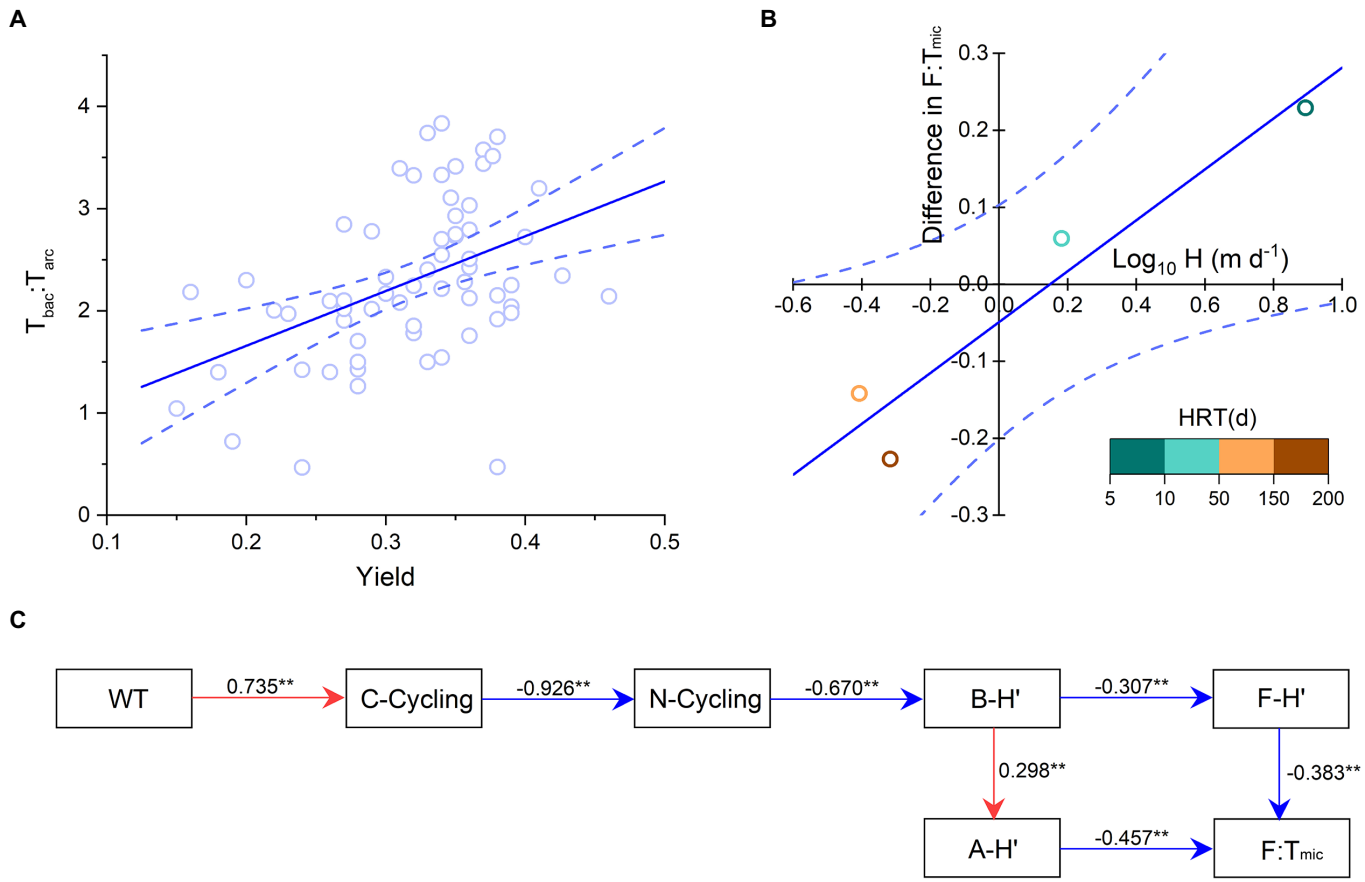
**(A)** Phytoplankton photosynthetic efficiency (yield) vs. the ratio of bacteria to archaea taxa-based Euclidean distance (T_bac_:T_arc_). The linear regression: y = 0.33x-0.05, adjusted *r^2^* = 0.88, *p* < 0.05. **(B)** Seasonal F:T_mic_ ratio difference vs. the hydraulic load (H). The linear regression: y = 5.36x + 0.59, adjusted *r^2^* = 0.19, *p* < 0.001. The different colors indicate different hydraulic retention times (HRT). The blue dotted lines indicate the 95% confidence intervals. **(C)** Structural equation modeling of microbial structure and function. The red and blue lines indicate the positive and negative relationships, respectively. The goodness of fit was acceptable [root mean square error of estimation (RMSEA) = 0.06, *p*-value for a test of close fit (P_CLOSE_) = 0.213, *n* = 78 independent samples]. ^**^ indicate the significance levels of 0.01. WT, water temperature; F:T_mic_, the ratio of functional pathway to microbial taxa-based Euclidean distance. A-H′, planktonic archaea community diversity; B-H′, planktonic bacteria community diversity; F-H′: functional diversity.

Mantel test indicated that the composition of functional pathways was related to 11 environmental factors, mainly including radiation, WT, and DSi. The community structure of planktonic archaea was related to 7 environmental factors, mainly including longitude, elevation, Ca/Sr, and DIP. The community structure of planktonic bacteria was relevant to eight environmental factors, mainly including radiation, WT, pH, DIC, and DSi (Figure 6A; Supplementary Figure 3). The relative abundances of C-, N-, P-, and S-cycling were correlated with dominant bacterial phyla (e.g., Proteobacteria, Actinobacteria, and Bacteroidetes) and their community diversity. The F:T_mic_ ratio was correlated with dominant archaea phyla (e.g., Thaumarchaeota and Nanoarchaeaeota) and their community diversity (Figure 6B). Co-occurrence networks showed that functional genes had tight contact with bacterial and archaeal community composition and were in a core position (Figure 6C), and planktonic bacterial community had higher relevancy with functional genes than archaeal community (Supplementary Figure 4). In addition, the average ratio of inflowing to reservoir waters was 1.11 for F and 1.22 for F:T_mic_, and both were significantly higher than 1 (*p* < 0.01 and *p* < 0.05, respectively, *t*-test); however, this case was not found for T_mic_ (*p* = 0.391, *t*-test, Figure 6D). Phytoplankton yield was positively correlated with T_bac_:T_arc_ (Figure 7A). The F:T_mic_ difference between August and December was positively correlated with Log_10_ transformed hydraulic load (Figure 7B). Structural equation modeling showed that WT was an important factor affecting EFS (Figure 7C).

## Discussion

### The relationship between microbial community structure and function

Phytoplankton and planktonic bacteria and archaea jointly drive the nutrient biogeochemical cycles in aquatic ecosystems (Arrigo, 2005; Fuhrman, 2009; Amin et al., 2021), and their community composition thus had a strong correlation with C-, N-, P-, and S-cycling functional genes (Figure 6C). The positive correlation between phytoplankton photosynthetic efficiency and T_bac_:T_arc_ implies that phytoplankton, as the primary producers, could constrain the bacterial and archaeal community assembly and their interaction (Figure 7A). The F:T_bac_ rather than F:T_arc_ was close to the F:T_mic_ (Figure 4), indicating that bacterial community was more relevant to the functional gene abundance than archaeal community. Therefore, planktonic bacteria could be more important to perform the microbial ecological functions in the reservoirs. Planktonic archaeal community had higher F:T_arc_, and their diversity synchronously changed with bacteria community diversity; therefore, they can also directly affect microbial EFS (Figure 7C). This implies that planktonic archaeal community, with strong interspecific interaction and low taxonomic variation, has an irreplaceable role in ensuring the stability of microbial ecological functions. Bacteria have been reported usually as the main microorganisms performing ecological functions under eutrophic conditions, while under oligotrophic conditions, archaea are the main functional microorganisms (Wuchter et al., 2006; Di et al., 2009).

### Effects of river damming on microbial ecological stability

Environmental changes are an important force driving the planktonic microbial species succession (Zhou et al., 2014; Amend et al., 2016; García-García et al., 2019; Xiao et al., 2021). In turn, planktonic bacteria and archaea have different responses to the environmental changes. In these reservoirs, planktonic bacterial community structure is mainly affected by WT and pH, while planktonic archaeal community composition is mainly influenced by geological and geographical factors. Major geological events and horizontal gene transfer have been reported to be relevant to the evolution of archaea (Yang et al., 2021; Gophna and Altman-Price, 2022). The composition of bacteria had tighter nexus with environmental factors than that of archaea (Figure 6A), implying that bacteria are easier to be affected by environmental variations. As such, bacteria and archaea are sensitive to different types of factors, finally leading to their ecological niche differentiation. In addition, archaea can take over the functions of bacteria under certain extreme conditions (Schleper, 2010; Wang et al., 2021). Microbial functional pathways have tight relation with the ambient parameters and are more sensitive to the environmental disturbance than bacterial and archaeal taxa in the reservoirs (Figure 6A), being consistent with the results of ocean study (Louca et al., 2016). For the reservoirs, water depth can not only regulate the spatial variation in microbial functional genes, but also restrict the planktonic bacterial community composition (Yang et al., 2020; Wang P. et al., 2022).

After river damming, water depth and hydraulic retention time increase, waters become clear, nutrients are impounded, and phytoplankton are apt to flourish (Maavara et al., 2020; Wang B. et al., 2022). An increase in WT can enhance phytoplankton growth, and phytoplankton consume CO_2_ and release O_2_ by photosynthesis, increasing water pH and DO. Thus, WT, Chl *a*, DO, and pH showed a tight relationship from each other in the reservoirs (Figure 6A). Phytoplankton photosynthesis mainly occurs in the euphotic layer, which can promote physical, chemical, and biological stratifications in the water profiles (Supplementary Figure 1). These stratifications create the contrasting redox environments between the surface and bottom waters, finally making the community composition of bacteria and archaea different in the water profiles, and the larger DO stratification, the more significant difference in their community composition (Yu et al., 2014; Nyirabuhoro et al., 2020; Yang et al., 2020). Correspondingly, matter cycling is different between the surface and bottom waters. On the bottom regulated by respiration, planktonic bacteria and archaea decompose organic matters to produce CH_4_ and N_2_O; SO_4_^2-^ can be used as an oxidant under insufficient oxygen conditions and is reduced to divalent S, which is apt to combine with heavy metals and then precipitate to the sediment (Goreau et al., 1980; Kuypers et al., 2018; Wang B. et al., 2022). On the surface dominated by photosynthesis, CH_4_ generated on the bottom can be re-oxidized to CO_2_ and release to the atmosphere (Vachon et al., 2019). Therefore, there are stratifications for the functional genes related to C-, N-, P-, and S-cycling in the reservoir profiles (Figure 3).

The significant changes in above physical and chemical factors after river damming finally result in obvious differences in the microbial EFS between rivers and reservoirs. Although there was not found significant difference in the community composition of bacteria and archaea between the inflowing and reservoir waters in general, relative compositions of their functional genes were significantly different, resulting in higher F:T_mic_ ratio in the former than in the latter (Figure 6D). All the evidence demonstrated that river damming can enhance the microbial EFS. In addition, the microbial EFS showed obviously different among the reservoirs, mainly due to their different hydrological conditions. It is well known that reservoir hydraulic load is an important factor governing the stratifications, nutrient cycling, and biological succession in reservoirs (Liang et al., 2019; Wang B. et al., 2022). Here, it was found that reservoir hydraulic load regulates the seasonal difference in microbial EFS (Figure 7B). In the daily regulated reservoirs (i.e., YT and DH), there are not significantly physical, chemical, and biological stratifications throughout the year due to the high water renewal rate, so they are river-liked reservoirs, where aquatic microorganisms are always living in a highly dynamic environment. The ambient conditions of these reservoirs were more strongly influenced by surface runoff in August than in December; correspondingly, microbial EFS was weaker in August than in December. In yearly regulated reservoirs (i.e., CST and LT), due to the low water renewal rate, microbial community structure and function were stratified, together with the physical and chemical stratifications in August, which finally shapes a unique strong microbial EFS in dam reservoirs. However, in December, the above stratifications disappeared mainly due to the homogenization of water temperature, and aquatic microorganisms have to re-adapt the situation *via* adjusting their community structure and function, which finally decreases microbial EFS. Therefore, microbial EFS is weaker in December than in August for the yearly regulated reservoirs (Figure 7B).

Studies on global ocean microbiome showed that microbial community composition is mostly affected by water temperature rather than other environmental factors or geography (Sunagawa et al., 2015). Similarly, this study found that WT is an important factor affecting the relationship between microbial community structure and functional pathways and then constraining the EFS (Figure 7C). Under the background of global warming, the variation in microbial ecological processes caused by the construction of numberless dam reservoirs should be paid more attention. This study focused on the impact of river damming on microbial EFS, while many other problems need to be further studied. For example, DSi was found as an important environmental factor influencing the microbial functions in co-occurrence networks, but the structural equation modeling cannot build the relevant pathway. It is well known that DSi not only reflect the geological background but also is closely related to phytoplankton community structure (Wang et al., 2016; Ran et al., 2022); however, we still do not know the underlying mechanism on the role of DSi in this study. The exploration on the microbial EFS is a basis for understanding the resistance of ecosystems, and more detailed ecological models need to be established for more precise assessment.

## Conclusion

This study first used the ratio of Euclidean-distance-normalized microbial function to taxa to estimate the influence of river damming on planktonic microbial EFS. The results indicated that river damming can enhance the planktonic microbial EFS. The microbial structure and function were stratified in the water profiles of dam reservoirs, together with the thermal and chemical stratifications, due to an increase in water depth and retention time. These changes after river damming resulted in the stronger microbial EFS in dam reservoirs than in rivers. Structural equation modeling demonstrated that water temperature was an important factor influencing the relationship between the microbial structure and function and then the EFS. In addition, the hydraulic load was found a main factor regulating the seasonal difference in microbial EFS among the reservoirs. This study will help to deepen the understanding of the relationship between microbial structure and function and provide a theoretical basis of assessing the ecological function change after the construction of river damming.

## Data availability statement

The datasets presented in this study can be found in online repositories. The names of the repository/repositories and accession number(s) can be found at: www.ncbi.nlm.nih.gov/sra/, PRJNA874581, PRJNA874587, PRJNA874586, and PRJNA874582.

## Author contributions

BW developed the idea. BW, SX, and C-QL optimized study. WL, NL, and MY performed the experiment and field work. WL and BW produced the first draft. All authors contributed to the article and approved the submitted version.

## Funding

This work was financially supported by the National Natural Science Foundation of China (U1612441) and the National Key Research and Development Program of China (2016YFA0601001).

## Conflict of interest

The authors declare that the research was conducted in the absence of any commercial or financial relationships that could be construed as a potential conflict of interest.

## Publisher’s note

All claims expressed in this article are solely those of the authors and do not necessarily represent those of their affiliated organizations, or those of the publisher, the editors and the reviewers. Any product that may be evaluated in this article, or claim that may be made by its manufacturer, is not guaranteed or endorsed by the publisher.

## Footnotes

^1^ www.wheata.cn
