# Supplementary material for: River damming enhances ecological functional stability of planktonic microorganisms
Source: Front Microbiol. 2022 Nov 30;13:1049120. doi: 10.3389/fmicb.2022.1049120 (PMC9749135; doi:10.3389/fmicb.2022.1049120)
Supplement: Supplementary file 1 [file Data_Sheet_1.PDF]

River damming enhances ecological functional stability of planktonic microorganisms

(Supplementary Information)

Wanzhu Li[1], Baoli Wang*[1, 2], Na Liu[1], Meiling Yang[1], Cong-Qiang Liu[1, 2], Sheng Xu[1]

[1] Institute of Surface-Earth System Science, School of Earth System Science, Tianjin University, Tianjin 300072, China.

[2] Tianjin Bohai Rim Coastal Earth Critical Zone National Observation and Research Station, Tianjin 300072, China.

***Corresponding author**: Baoli Wang (Email: baoli.wang@tju.edu.cn), Institute of Surface-Earth System Science, School of Earth System Science, Tianjin University, Tianjin 300072, China. Tel: +86 (0)22 27405053; Fax: +86 (0)22 27405051.

Supplementary Table

Table S1. The detailed information of sampling sites in the studied reservoirs.

Date	Reservoir	Number	Type	Depth (m)	Longitude °E	Latitude °N
2019-8	Dahua (DH)	DH-0	Inflowing water	0	107.5401	23.9751
		DH-1	Reservoir	0	107.9031	23.7246
		DH-2-0	Reservoir	0	107.9784	23.7284
		DH-2-5	Reservoir	5	107.9784	23.7284
		DH-2-10	Reservoir	10	107.9784	23.7284
		DH-2-15	Reservoir	15	107.9784	23.7284
		DH-2-25	Reservoir	25	107.9784	23.7284
		DH-3	Released water	0	107.9941	23.7343
	Yantan (YT)	YT-0	Inflowing water	0	107.4154	24.5686
		YT-1	Inflowing water	0	107.2760	24.1635
		YT-2	Reservoir	0	107.4770	24.0882
		YT-3	Reservoir	0	107.5085	24.0783
		YT-4-0	Reservoir	0	107.5123	24.0440
		YT-4-05	Reservoir	5	107.5123	24.0440
		YT-4-10	Reservoir	10	107.5123	24.0440
		YT-4-15	Reservoir	15	107.5123	24.0440
		YT-4-30	Reservoir	30	107.5123	24.0440
		YT-4-50	Reservoir	50	107.5123	24.0440
		YT-5	Released water	0	107.5175	24.0265
	Longtan (LT)	LT-0	Inflowing water	0	105.9509	25.0737
		LT-1	Inflowing water	0	105.5285	24.8042
		LT-2	Reservoir	0	107.0179	25.1013
		LT-3	Reservoir	0	107.0143	25.0193
		LT-4-0	Reservoir	0	107.0392	25.0281
		LT-4-5	Reservoir	5	107.0392	25.0281

		LT-4-10	Reservoir	10	107.0392	25.0281
		LT-4-15	Reservoir	15	107.0392	25.0281
		LT-4-30	Reservoir	30	107.0392	25.0281
		LT-4-60	Reservoir	60	107.0392	25.0281
		LT-4-80	Reservoir	80	107.0392	25.0281
		LT-4-140	Reservoir	140	107.0392	25.0281
		LT-5	Released water	0	107.0730	25.0144
	Chaishitan (CST)	CST-1	Inflowing water	0	103.4794	24.9400
		CST-2	Reservoir	0	103.3852	25.0069
		CST-3-0	Reservoir	0	103.3371	24.9954
		CST-3-5	Reservoir	5	103.3371	24.9954
		CST-3-10	Reservoir	10	103.3371	24.9954
		CST-3-15	Reservoir	15	103.3371	24.9954
		CST-3-30	Reservoir	30	103.3371	24.9954
		CST-3-55	Reservoir	55	103.3371	24.9954
		CST-4	Released water	0	103.3295	25.0013
2019-12	Dahua (DH)	DH-0	Inflowing water	0	107.5401	23.9751
		DH-1	Reservoir	0	107.9031	23.7246
		DH-2-0	Reservoir	0	107.9784	23.7284
		DH-2-5	Reservoir	5	107.9784	23.7284
		DH-2-10	Reservoir	10	107.9784	23.7284
		DH-2-15	Reservoir	15	107.9784	23.7284
		DH-2-25	Reservoir	25	107.9784	23.7284
		DH-3	Released water	0	107.9941	23.7343
	Yantan (YT)	YT-0	Inflowing water	0	107.2760	24.1635
		YT-1	Inflowing water	0	107.4154	24.5686
		YT-2	Reservoir	0	107.4770	24.0882
		YT-3	Reservoir	0	107.5085	24.0783
		YT-4-0	Reservoir	0	107.5123	24.0440

	YT-4-05	Reservoir	5	107.5123	24.0440
	YT-4-10	Reservoir	10	107.5123	24.0440
	YT-4-15	Reservoir	15	107.5123	24.0440
	YT-4-30	Reservoir	30	107.5123	24.0440
	YT-4-50	Reservoir	50	107.5123	24.0440
	YT-5	Released water	0	107.5175	24.0265
Longtan (LT)	LT-0	Inflowing water	0	105.9509	25.0737
	LT-1	Inflowing water	0	105.5285	24.8042
	LT-2	Reservoir	0	107.0179	25.1013
	LT-3	Reservoir	0	107.0143	25.0193
	LT-4-0	Reservoir	0	107.0392	25.0281
	LT-4-5	Reservoir	5	107.0392	25.0281
	LT-4-10	Reservoir	10	107.0392	25.0281
	LT-4-15	Reservoir	15	107.0392	25.0281
	LT-4-30	Reservoir	30	107.0392	25.0281
	LT-4-60	Reservoir	60	107.0392	25.0281
	LT-4-80	Reservoir	80	107.0392	25.0281
	LT-4-140	Reservoir	140	107.0392	25.0281
	LT-5	Released water	0	107.0730	25.0144
Chaishitan (CST)	CST-1	Inflowing water	0	103.4794	24.9400
	CST-2	Reservoir	0	103.3852	25.0069
	CST-3-0	Reservoir	0	103.3371	24.9954
	CST-3-5	Reservoir	5	103.3371	24.9954
	CST-3-10	Reservoir	10	103.3371	24.9954
	CST-3-15	Reservoir	15	103.3371	24.9954
	CST-3-30	Reservoir	30	103.3371	24.9954
	CST-3-55	Reservoir	55	103.3371	24.9954
	CST-4	Released water	0	103.3295	25.00133

Table S2. The main features of the studied reservoirs in the Pearl River basin. The full names of the reservoirs refer to the text.

Reservoir	Hydraulic retention time (d)	Average depth (m)	Regulation type
CST	104.3	40.8	Yearly
LT	192.5	92.7	Yearly
YT	22.0	33.5	Daily
DH	5.6	43.9	Daily

Supplementary Figure

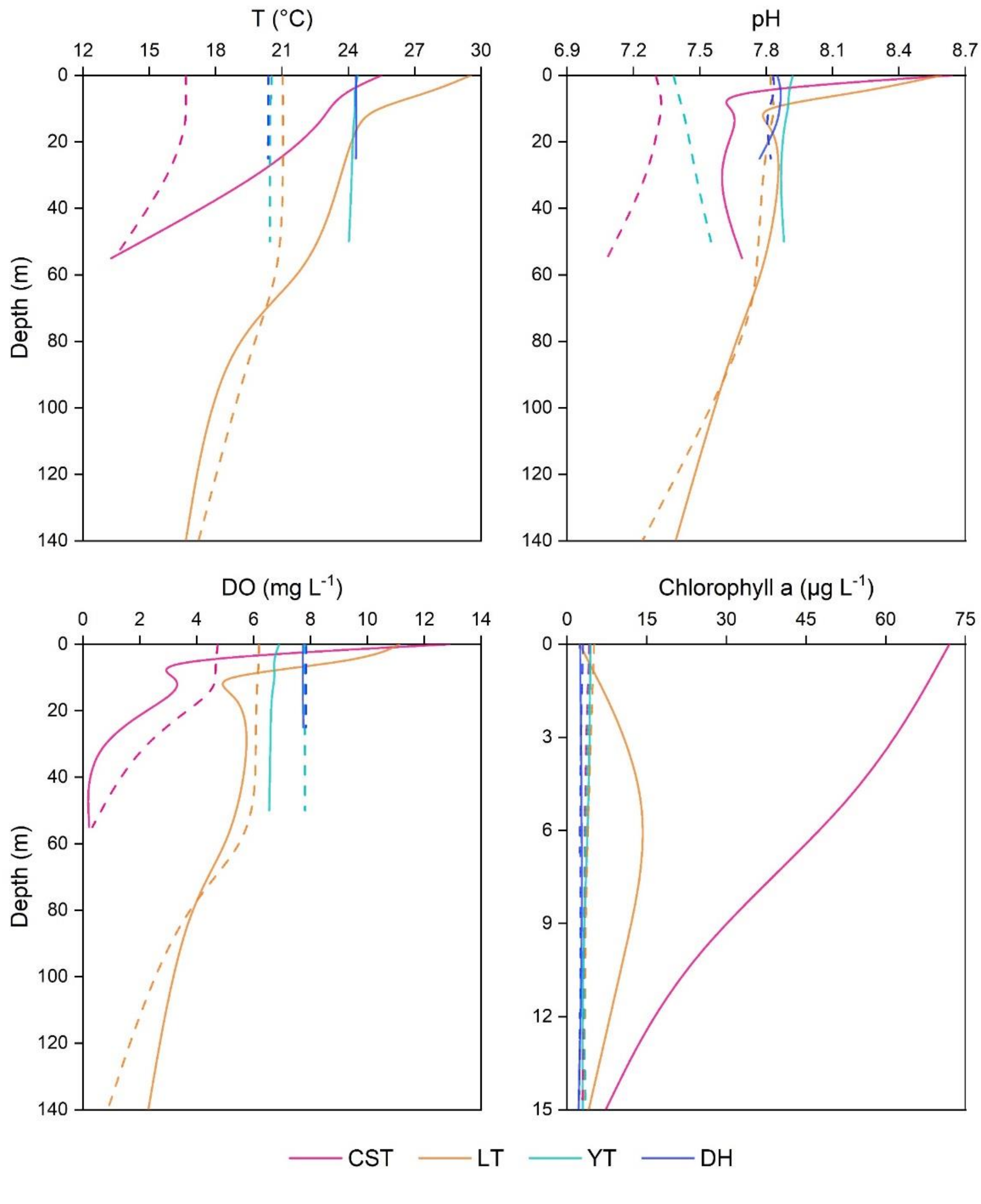

Figure S1. The parameter variations in the reservoir profiles. The different colors indicate different reservoirs, and their full names refer to the text. The solid and dotted line represent sampling in August and December, respectively. T, water temperature; DO, dissolved oxygen concentration.

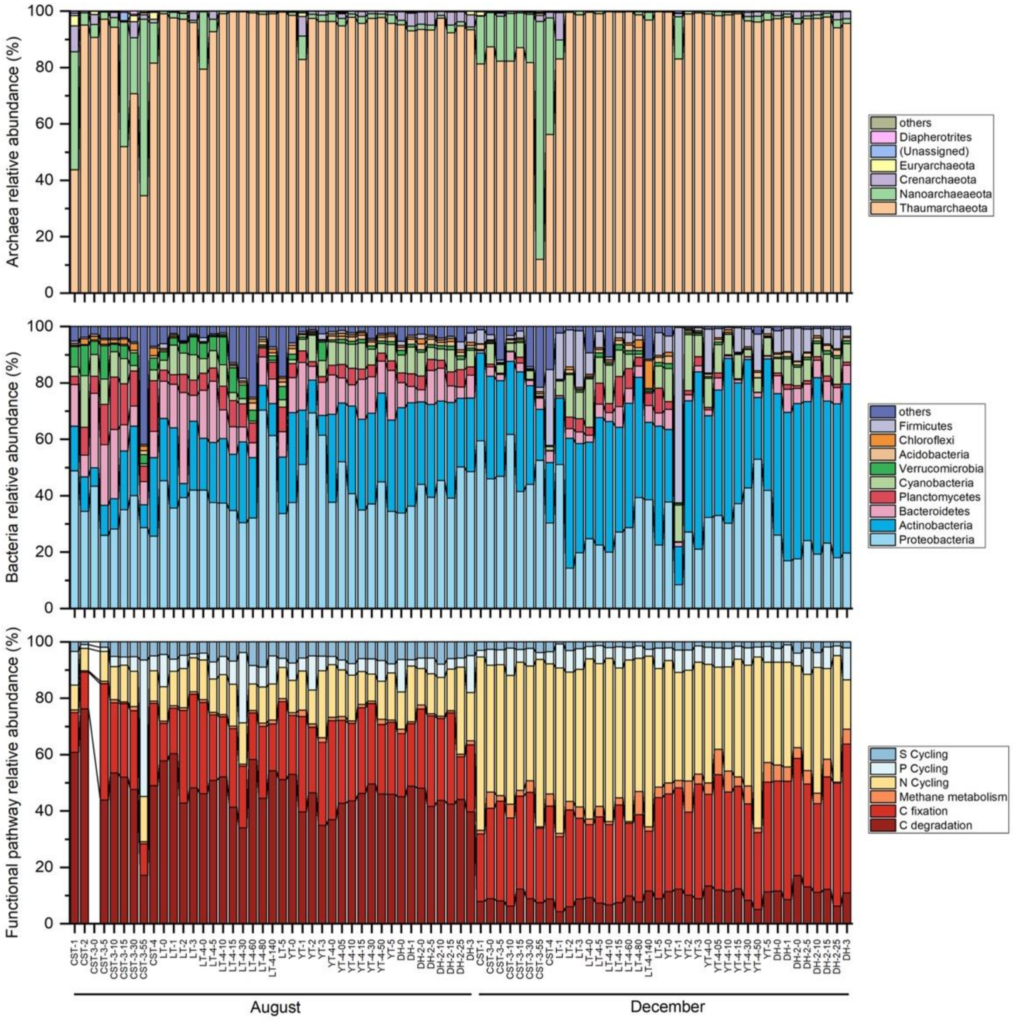

Figure S2. The spatio-temporal distribution of planktonic archaea and bacteria community composition and relative abundance of functional pathway in the studied reservoirs. Taxonomic groups with a relative abundance less than 1% were integrated as “others”. The full names of the reservoirs refer to the text. The functional data of CST-3-0 in August were missing.

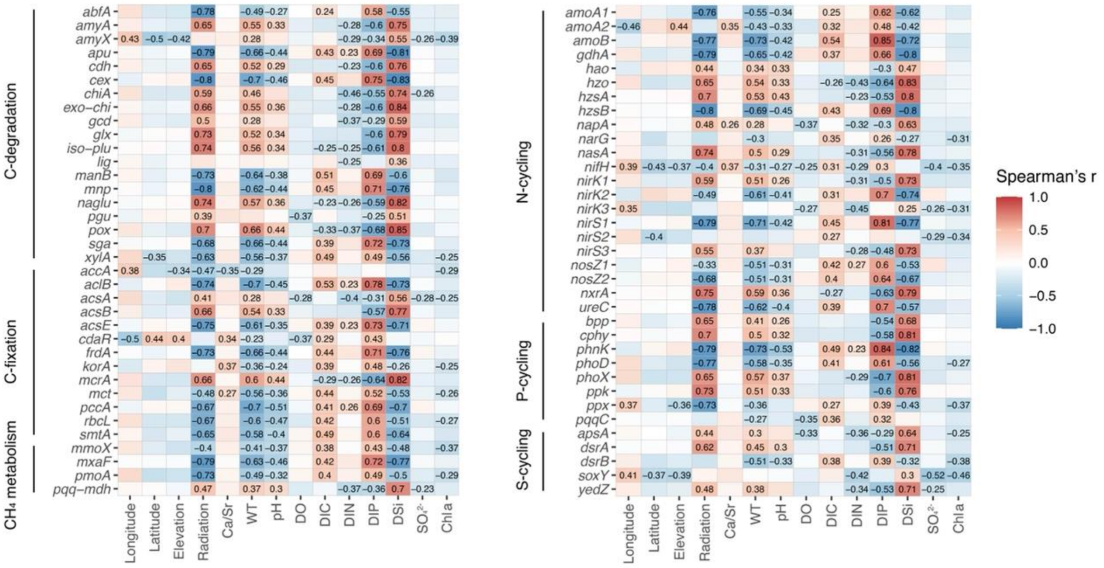

Figure S3. Correlation between functional gene and environmental factors. Pairwise comparisons are displayed with a color gradient to denote Spearman's correlation coefficients. WT, water temperature; DO, dissolved oxygen; DIC, dissolved inorganic carbon; DIN, dissolved inorganic nitrogen; DIP, dissolved inorganic phosphorus; DSi, dissolved silicon; Chl *a*: chlorophyll a concentration.

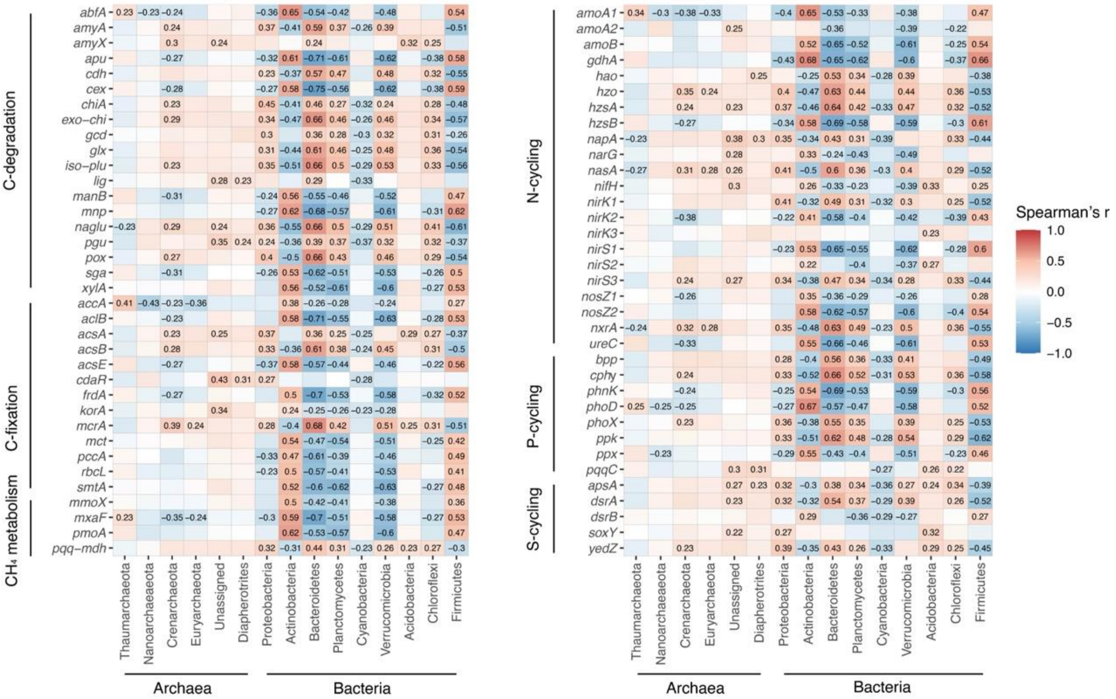

Figure S4. Correlation between functional gene and archaea and bacteria community composition. Pairwise comparisons are displayed with a color gradient to denote Spearman's correlation coefficients.
